# Criteria for Teaching Performance in Psychology: Invariance According to Age, Sex, and Academic Stage of Peruvian Students

**DOI:** 10.3389/fpsyg.2021.764081

**Published:** 2021-10-26

**Authors:** Aldo Bazán-Ramírez, Juan Carlos Pérez-Morán, Brando Bernal-Baldenebro

**Affiliations:** ^1^Medicine School, César Vallejo University, Herrera, Peru; ^2^Institute of Educational Research and Development, Autonomous University of Baja California, Ensenada, Mexico

**Keywords:** teaching performance, psychology professor, didactic interaction, invariance, Peruvian students

## Abstract

The use of scales to assess the performance of professors from the students' standpoint is a generalized practice in higher education systems worldwide. The purpose of this study is to analyze the factorial structure and measure the invariance of the Scale of Teaching Performance of the Psychology Professor (EDDPsic) among groups according to gender, age, and academic stage. The sample of participants was composed of 316 Psychology students from the fourth and sixth semesters (basic cycles), and from the eighth and tenth semesters (disciplinary-professional cycles) of two renowned public universities in Lima, Peru. Two hundred and thirty-one participants were women (73%), and the mean age of students was 21.5 years old (*SD* = 2.37). The measurement invariance of the scale in the three study variables was underpinned by a multigroup confirmatory factor analysis (MGCFA) conducted using a five-factor model that showed the best fitness indices. It is concluded that significant differences in measuring teaching performance areas of the professor depend on the students' age difference and on their academic stage (to attend the disciplinary-professional cycles).

## Introduction

The use of self-reporting questionnaires, both by the professors themselves and by the students, has been a generalized practice to identify and assess the behavior of professors and their teaching attributes during didactic interactions (Simpson and Bester, 2016; Darwin, 2017; Doménech-Betoret, 2018). The scales used by PISA (Organisation for Economic Co-operation Development., 2013, 2019) to assess both the teaching practice and the quality of learning are practical examples of this kind of instruments.

The presented research is not an assessment of teaching performance from the students' standpoint per se. Traditionally, having students assess their professors' performance has served as a mechanism to evaluate teaching in several universities worldwide, and it has to do with administrative-academic management; it can even serve as an accountability mechanism for comparative assessment of academic programs and universities (Boysen, 2016; Darwin, 2017). Nonetheless, several opposing views have arisen regarding whether an assessment from students does represent a valid perception of didactic interactions and the efficiency of teaching (Gray and Bergmann, 2003; Boysen, 2016; Hornstein, 2017; Newton et al., 2019).

In this study, assessing the professor's behavior in various areas of didactic performance aims to identify opportunities during their class or practice to improve their own didactic practice, rather than it being a form of administrative management. From this standpoint, self-reporting instruments seek to identify patterns or the occurrence frequency of certain behaviors during didactic interactions (Bazán and Velarde, 2021).

The substantive theory that guides the suitability of identifying behaviors in one of the components of the didactic triad (who teaches, who learns, and what is being taught and learned) is based on Kantor's (1959) interbehavioral psychology. According to this approach, psychological events represent interactions in a multifactor field with elements or factors that are functionally interrelated with one another (Kantor and Smith, 1975; Fryling et al., 2011; Hayes and Fryling, 2018). In so doing, special classroom conditions and the professor's behavior (and their strategies) can be identified and analyzed in the context of didactic interactions (in which interbehavioral fields are configured), as well as any of the other multiple factors that affect interbehavior (Kantor and Smith, 1975). According to Kantor:

Interbehavioral psychology places teachers in proper perspective when they are considered as part of the setting of educational situations. They provide specific favorable or unfavorable circumstances for the advantage or disadvantage of acquiring reactions, accomplishing tasks, and setting up opportunities for development. Not only are teachers peripheral factors in learning situations, but they can only operate in conjunction with background and foreground features surrounding the modification of the taught (Kantor, 1975, pp. 317–318).

In this approach, a didactic performance model was proposed from an interconductual psychology perspective, for the practice and exercise of science teaching (Carpio et al., 1998; Irigoyen et al., 2011, 2016; Silva et al., 2014), in other words, these categories can be used in the analysis of didactic interactions in other scientific disciplines and at different grades and levels of education. Carpio et al. (1998) proposed a model for the sciences professor's performance with five performance categories or criteria: Cognitive Exploration, Criteria Explanation, Illustration, Practice, and Evaluation. Silva et al. (2014) would later expand said model to seven teaching performance categories or criteria: Planning, Competence Exploration, Criteria, Illustration, Practice Supervision, Feedback, and Evaluation.

Derivations of this model have been made for the study of didactic performance in didactic interactions and in the teaching and learning of science at Bachillerato level—equivalent to High-School or K10–K12 levels (Velarde and Bazán, 2019; Ávila, 2020), as well as in teaching of psychology (Silva et al., 2016; Bazán and Velarde, 2021).

Several areas or criteria defining different actions by the professor during didactic interactions (i.e., classes or practices) can be identified based on this substantive theory on the Psychology professor's performance. Solely for analysis purposes, it is possible to identify the separate actions carried out by teachers during a didactic interaction. To that end, different procedures can be followed that have proven to be useful for understanding and explaining teaching practices and instructional processes in the University context.

With respect to dimensions of the assessment of teaching performance and invariance sources, the literature on this subject shows a wide diversity of dimensions and constructs used to identify and assess the professors' performance and the teaching–learning process according to the students. Several aspects of teaching–learning have been included as dimensions of measurement, conceiving their multidimensional structure, whereas contextual factors have also been considered as variables that may influence the students' assessments.

Hereunder, we have assembled the main dimensions of teaching, which have been specified for assessment from the students:

- A first set of dimensions to be assessed focuses on the professor–student interaction and classroom management: Conditions in the classroom and group relationships (Marsh, 1984; García-Gómez et al., 2017; Nasser-Abu, 2017; Chan, 2018; Bell et al., 2019).
- A second set of dimensions has to do with teaching specifics: Methods, approaches, contents, and teaching activities, as well as the correlation between theoretical teaching and practical components, applied to real situations (Entwistle, 2007; García-Gómez et al., 2017; König et al., 2017; Nasser-Abu, 2017; Chan, 2018).
- The third approach encompasses the characteristics of teaching planning and organization, which involve planning lessons, laying out objectives to be attained, and the harmonious organization of the class learning process (Marsh, 1984; García-Gómez et al., 2017; König et al., 2017; Nasser-Abu, 2017; Krijgsman et al., 2019).
- A fourth dimension includes features of curriculum coverage and the complexity of teaching–learning structuring (type of required cognitive demand). It also refers to the assignment of tasks and the provision and use of educational materials that contribute to the learning process (Marsh, 1984; Entwistle, 2007; Grammatikopoulos et al., 2015).
- The fifth dimension is the learning assessment process, and has to do with examinations, formative assessment, feedback from students, and reflections on the teaching practice (Marsh, 1984; Entwistle, 2007; Grammatikopoulos et al., 2015; García-Gómez et al., 2017; Nasser-Abu, 2017; Bell et al., 2019; Jellicoe and Forsythe, 2019; Krijgsman et al., 2019).
- To a lesser extent, teacher support to students during the learning process, motivation, and sharing learning and self-regulation strategies with them have been regarded as dimensions of higher education teaching (Entwistle, 2007; Bell et al., 2019).

While it is true that researchers differ when choosing from a variety of constructs to refer to variables or aspects of teaching and of the professors' behavior during class, many of these studies have addressed several contextual and student characteristics as control variables to assess whether these variables differentially affect how students evaluate teaching and their professor.

One first aspect refers to attributes of the students, including age, sex, educational background, desirability, and expectations and prior interest (Marsh, 1984; van de Grift et al., 2016; Feistauer and Richter, 2018). Another aspect is that some details of the professor being evaluated have been included as control sources in the students' assessment, for example, their sex, expertise, quality, and hierarchy, their personal traits and sense of humor, and how challenging their class seems (Cochran et al., 2003; Grammatikopoulos et al., 2015; Scherer and Gustafsson, 2015; Chan, 2018; Feistauer and Richter, 2018). Apparently, the more efficient a professor is, the worst assessments they get from students (Eouanzoui and Jones, 2017).

An invariance analysis and a multilevel hierarchical analysis have been conducted as a methodological strategy for data analysis to control possible sources of invalidity when measuring assessments from students regarding teaching and their professors' behavior. Nonetheless, invariance analyses through multigroup approaches for measuring and validating multidimensional constructs are still in development (Byrne, 2008; Milfont and Fischer, 2010; Hirschfeld and Von Brachel, 2014; Marsh et al., 2020); they possess a great diversity of strategies and statistical programs, and factorial analyses through structural equation modeling (SEM) have been favored.

Other aspects have been included for these analyses, such as: Validity of measurement constructs (Hornstein, 2017), the professor's and the students' sex (Boring, 2015; Eouanzoui and Jones, 2017), their semester or academic stage and type of class (Kalender, 2015), a same class taught at different academic semesters or years (Marsh and Hocevar, 1984), the grades obtained by students and the professor's qualification (Üstünlüoglu and Güngör-Culha, 2012; Spooren et al., 2017), or the same degree program or discipline taught at different institutions (Müller et al., 2017).

Generally, these studies have addressed various disciplines, but little is known about the assessments made by Psychology students regarding teaching performance criteria in the didactics of Psychology classes, and about construct validity and invariance of such assessments according to various student variables, for example, the sex, age, and academic stage of students.

The purpose of this study is to analyze the structure and factorial invariance of the self-reporting measurement by undergraduate Psychology students from two public universities in Lima, Peru, with respect to five competences (criteria) of didactic performance of professors during Psychology classes. To this end, the teaching performance criteria categories were adapted (Irigoyen et al., 2011; Silva et al., 2014) by specifying five teaching performance criteria: Competence Exploration, Criteria Explanation, Illustration, Feedback, and Evaluation. These categories will be outlined in the Method section.

According to the objective of this work, it tested a hypothetical model of convergent and divergent analysis of five criteria of teacher didactic performance, assessed by psychology students through self-report with the Scale of Teaching Performance of the Psychology Professor (Bazán and Velarde, 2021), as well as its measurement invariance according to sex, age, and academic stage. Factorial invariance was calculated including three variables that might indicate differences in the teaching performance assessment from students in didactic interactions: age, sex, and academic stage of the students. These variables have been reported in the current literature as control sources of factorial invariance of the instruments to identify student and professor performance assessment (Kalender, 2015; Kalender and Berberoglu, 2019). A multigroup confirmatory factor analysis (MGCFA) procedure was followed, closely related to the analysis models proposed by Byrne (2008) and Hirschfeld and Von Brachel (2014).

A second hypothesis was the existence of significant differences in the evaluation of the didactic performance of the teacher, according to sex, age groups, and academic level (stage) of the psychology students. To date, little is known about possible differences according to the age of the student body in the appraisal of teaching in the context of University education, but differences in students' appraisal of teaching according to their level of advancement in their studies have been reported (Marsh and Hocevar, 1984; Kamran et al., 2012; Kalender, 2015; Müller et al., 2017; Kitto et al., 2019; Mocanu et al., 2021; Pérez-Villalobos et al., 2021). Similarly, differences have been reported according to the gender of the student body, with respect to the students' assessment of teaching and the performance of their teachers, in the context of higher education (Boring, 2015; Boring et al., 2016; Potvin and Hazari, 2016; Eouanzoui and Jones, 2017; Heffernan, 2021; Kreitzer and Sweet-Cushman, 2021; Valencia, 2021).

## Methods

### Participants and Procedure

Participants were 316 University students attending different terms of the Psychology course (4th, 6th, 8th, and 10th semesters): 239 students from the Federico Villarreal University and 77 students from the National Major University of San Marcos. Convenience sampling was conducted; 231 participants were women (73%) and 85 were men (27%), and the mean age of participants was 21.5 years old (SD = 2.37). An important consideration for the disproportionate number between female and male participants is that the psychology student body is essentially female-majority. While the sampling was not probabilistic and depended largely on the assignment of the authorities themselves, the gender configuration shows this difference in the composition of the student population of psychology majors.

In CFA and factorial invariance test the sample size can influence the goodness-of-fit indicators (Dimitrov, 2010) and the power of the test (Putnick and Bornstein, 2016). However, different authors propose the possibility of performing analyses for SEM and factorial invariance tests with small samples (Bentler and Yuan, 1999; Chen et al., 2008; *N* < 100). Increasing the sample size in turn produces, in the chi-square test (χ^2^), an increase in the rejection of the null hypothesis. Likewise, the invariance measurement tests are affected by the changes in the chi-square caused by the increase in the sample, so it is recommended to evaluate the fit with absolute fit indices, such as the RMSEA to correct for the over-rejection of models using small samples (Cheung and Rensvold, 2002).

There is currently no agreement among experts on the minimum sample size for this type of study (Thompson, 2000). There is also no agreement on the number of cases per variable. For convergent and divergent validation studies of constructs, analyses can be conducted with 10 or 20 subjects per variable (Schumacker and Lomax, 1996). Bentler and Chou (1987) suggest a minimum ratio of five cases per latent variable when there are multiple indicators, as in the case of the present study where there are five factors.

Table 1 shows the sample of study participants per sex and term. It is noteworthy that most participants (166; 52.5%) were currently studying the intermediate level terms (fourth and sixth semesters), whereas the rest (47.5%) attend the disciplinary-professional terms (eighth and tenth semesters). The subjects of the fourth and sixth cycles correspond to intermediate subjects that are fundamental to the discipline of psychology, both theoretically and methodologically. On the other hand, the final level of disciplinary progress corresponds to the eighth and tenth cycles; at this level, the subjects involve knowledge and skills for professional practice and applications of psychology in various fields.

**Table 1:** Sample of study participants per sex and course term.

**Variable**		**Course term (semester)**				
		**4th (%)**	**6th (%)**	**8th (%)**	**10th (%)**	**Total (%)**
Sex	Men	30 (9.5%)	18 (5.7%)	22 (7.0%)	15 (4.8%)	85 (27%)
	Women	84 (26.5%)	34 (10.8%)	68 (21.5%)	45 (14.2%)	231 (73%)
	Total (%)	114 (36.0%)	52 (16.5%)	90 (28.5%)	60 (19.0%)	316 (100%)

For this study, the following subjects were evaluated in the 4th cycle: Construction of Psychological Tests, Research Design, Psychodynamics, and Cognitive Theory, while in the 6th cycle, Clinical Psychology, Clinical and Health Psychology, and Experimental Analysis of Behavior II were evaluated.

Likewise, in the eighth cycle the following subjects were included: Human Resources and Knowledge Management, Educational Psychology II, Psychopathology II, and Research Seminar. For the tenth cycle, the following subjects were evaluated: Behavioral Analysis Applied to Education II, National Defense (Social Psychology), Intervention Strategies and Preventive Programs in Clinical Psychology.

### Instrument

For the purposes of this study, we devised the Scale of Teaching Performance of the Psychology Professor (EDDPsic) based on different well-established theoretical models in the field of teaching performance assessment (Irigoyen et al., 2011; Silva et al., 2014). The instrument used was an adaptation and redesign of new items, by a panel of Peruvian experts, of the instrument previously validated with Mexican psychology students by Bazán and Velarde (2021). That is, based on the study referred to as direct antecedent, improvements were made to this new version. To that end, a committee of experts was assembled to verify the relevance and design of the items of the EDDPsic. Likewise, the departments in charge of managing the research projects at both participating public universities reviewed the instruments and authorized their application on the selected sample of students.

The EDDPsic is composed of 18 items arranged into five didactic performance factors or criteria: Competence Exploration (ECO), Criteria Explanation (ECR), Illustration (ILU), Feedback (RTA), and Evaluation (EVA). The items' format sets out four answer options: Never, Almost never, Almost always, and Always. Furthermore, two of the 18 items of the Self-reporting questionnaire on didactic interactions validated by Bazán and Velarde (2021) were used unchanged for this scale. Table 2 lists the items and factors that constitute the EDDPsic.

**Table 2:** Items of the scale of teaching performance of the psychology professor (EDDPsic).

**EDDPsic factors**	**Item ID**	**Items per teaching performance**
Competence exploration (ECO)	des_1	The professor assessed our prior knowledge at the beginning of the term, either in writing or orally.
	des_2	The professor examined our existing abilities at the beginning of the term.
	des_3	The professor examined my knowledge on the class at the beginning of each session.
Criteria explanation (ECR)	des_4	The professor explains the criteria and requirements needed to perform a class practice.
	des_5	The professor indicates which learning goals are to be achieved throughout the term.
	des_6	The professor indicates what we have to learn during each session.
	des_7	In terms of the class, the professor indicates what criteria are expected from professionals with my pursued degree.
	des_8	The professor highlights what necessary abilities must we develop throughout the class.
Illustration (ILU)	des_9^*^	*The professor thoroughly explains the session's topic*.
	des_10	Concerning a solution, the professor describes what constitutes the solution, and when and why to apply said solution.
	des_11	The professor outlines wrong solutions to a problem before revealing the right solution.
Feedback (RTA)	des_12	The professor corrects our performance during class activities.
	des_13^*^	*The professor teaches several ways to comply with the achievement criteria of their class activities*.
	des_14	After outlining the class and practice, the professor provides suggestions to improve our performance.
	des_15	Concerning a solution, the professor describes the procedure that we follow and tells us how we can improve.
Assessment (EVA)	des_16	The professor applies examinations and provides a solution to practical problems derived from the class.
	des_17	The professor assesses the students according to the learning objectives shown at the beginning of the term or to those found in the syllabus.
	des_18	The teaching assessment system is suitable to measure our knowledge and abilities attained throughout the class.

*Source: Own work*.

* *Items used unchanged from the original version by Bazán and Velarde (2021)*.

### Data Collection

For the data collection process, classes were first selected for application of the instrument in the classrooms according to instructions from the directors of the participating universities. Then, each of the professors of the chosen classes was contacted to explain the rationale behind the study and to request their authorization to apply the EDDPsic to their students from the previous semester. Finally, students and professors were given informed consent and the application schedule was agreed upon. Efforts were made to ensure that all participating students answered all the items of the instrument.

### Data Analysis

During the period January–May 2019, databases were created from the collected answers and purged. The database was purged by removing cases with missing values and cases with a General Index (IG) deemed atypical (IG < Q1 – 1.5 IQR; IG > Q3 + 1.5 IQR). Then, statistical analyses were carried out to obtain: (a) descriptive statistics, (b) normal distribution univariate indices, (c) internal consistency indices, (d) joint association degree indices among variables, (e) construct validity evidence of the internal structure, (f) factorial invariance evidence according to the sex, age, and academic stage variables, and, lastly, (g) a comparative analysis of mean scores among participants from different groups.

All statistical analyses were performed running the open-source software RStudio version 1.4, using mainly the dplyr, psych, lavaan, and semTools packages while following the recommendations from Hirschfeld and Von Brachel (2014). Furthermore, the same analyses were applied using the IBM SPSS and AMOS software version 23 to support the obtained data.

Descriptive statistics for mean, median, standard deviation and error, and kurtosis and skewness were obtained for each item. To validate the assumption of a normal univariate data distribution, the kurtosis and skewness values of the IG and of each item were assessed. Likewise, the Lilliefors-corrected Kolmogorov-Smirnov goodness-of-fit test was applied. Criteria for acceptance of normal data distribution were kurtosis and skewness values approaching 0 (Tabachnick and Fidell, 2012) and *p* < 0.03 for the Lilliefors-corrected Kolmogorov-Smirnov goodness-of-fit test (Gerard and Leland, 1986). The Cronbach's alpha (α), Rho ordinal standardized alpha (ρ), and McDonald's Omega (ω) coefficients were calculated to obtain internal consistency indices. Internal consistency criteria for the scale were determined as α*-* and ρ*-*values ≥0.70 (Hair et al., 2019) and a ω-value ≥0.80 (Nájera-Catalán, 2019).

In order to obtain construct validity evidence of the internal structure of the scale, the polychoric correlation matrix of the data was verified (see Supplementary Table S1) along with the degree of joint association among variables. To that end, the Bartlett sphericity test and the Measure of Sampling Adequacy (MSA) by Kaiser-Meyer-Olkin (KMO) were applied. A joint association degree among variables was deemed acceptable under the criteria *p* ≤ 0.50 for the Bartlett sphericity test (Hair et al., 2019) and an MSA-value ≥0.70 for KMO (Hill, 2011; Hair et al., 2019).

Afterwards, Confirmatory Factor Analyses (CFA) were performed for a five-factor model of the EDDPsic, based on the recommendations from Bazán and Velarde (2021). The fitness of models was assessed considering the recommendations from Hu and Bentler (1999). Calculated indices were: Comparative Fit Index (CFI), Non-Normed Fit Index (NNFI), Standardized Root Mean Square Residual (SRMR), and Root Mean Square Error of Approximation (RMSEA). The value criteria to deem fitness acceptable for the model were: Comparative Fit Index and NNFI approaching 0.95, SRMR approaching or <0.08, and RMSEA approaching or <0.06.

A MGCFA was performed with the purpose of obtaining evidence of factorial invariance according to the sex, age, and academic stage variables. For the analyses according to sex, cases were divided into men and women; for age, into 21 years old or younger and 22 years old or older; and for academic stage, into disciplinary stage terms (4th and 6th semesters) and final stage terms (8th and 10th semesters). The recommendations from Byrne et al. (1989); Vandenberg and Lance (2000); Byrne (2008), Dimitrov (2010), and Milfont and Fischer (2010) were followed during the execution and interpretation of techniques and tests related to factorial invariance.

A sequential restrictions procedure was followed, so comparisons were made using models with increasingly restrictive parameters. The five-factor model from the previous stage was taken as a basis and the groups were compared through the configurational (same structure among groups), weak (same factorial loads among groups), strong (same item intercepts among groups), and strict (same error variance among groups) models. The criteria to consider factorial invariance among models as adequate was a non-significant value difference (*p* ≥ 0.05) in the chi-square test (Δχ^2^), a CFI difference below −0.01 (ΔCFI ≤ 0.01), and an RMSEA value approaching or lower than 0.06 (Vandenberg and Lance, 2000; Cheung and Rensvold, 2002; Dimitrov, 2010; RMSEA ≤ 0.06).

Lastly, comparative analyses were conducted on the mean scores of participants from different groups. Said comparative analyses were applied to the groups that showed, at the least, evidence of factorial invariance of the strict model, that is, those with invariant intercepts and factorial loadings. Should evidence not be found, interpretation of comparisons among means from participants is susceptible to bias (Dimitrov, 2010; Milfont and Fischer, 2010). Comparisons of mean scores were analyzed based on the results from the variance analysis test (ANOVA). The criterion to consider the difference among groups as valid was obtaining a *p* ≥ 0.05 value in the ANOVA test (Creswell, 2012). In addition, the effect size was calculated using the eta-squared (η^2^) coefficient; to that end, the recommendations from Richardson (2011); Lakens (2013), and Funder and Ozer (2019) were followed for calculating and interpreting the results from said coefficient.

## Results

### Descriptive and Preliminary Analyses

Mean, standard deviation and error, and skewness and kurtosis values were calculated for the IG and for each item of the EDDPsic. The means and standard deviations of the items yielded values ranging from 2.63 to 3.19, and from 0.62 to 0.94, respectively. The means for most of the items and the IG indicate that, according to participants, professors exercise observed behaviors with an almost always frequency. Results from the Lilliefors-corrected Kolmogorov-Smirnov test, as well as the skewness and kurtosis values for each item allow to consider the univariate normal distribution assumption as accepted. Descriptive statistics for each item and IG are shown in Table 3.

**Table 3:** Descriptive statistics for each item and IG.

**Item**	**Mean**	**Standard deviation**	**Asymmetry**	**Kurtosis**	**Standard error**
des_1	2.63	0.94	−0.13	−0.88	0.05
des_2	2.79	0.77	−0.27	−0.26	0.04
des_3	2.72	0.77	−0.01	−0.54	0.04
des_4	3.10	0.67	−0.51	0.59	0.04
des_5	3.19	0.69	−0.38	−0.41	0.04
des_6	3.13	0.67	−0.35	−0.02	0.04
des_7	3.10	0.68	−0.19	−0.63	0.04
des_8	3.17	0.65	−0.32	−0.13	0.04
des_9	3.14	0.70	−0.42	−0.16	0.04
des_10	3.17	0.62	−0.13	−0.53	0.04
des_11	2.94	0.75	−0.18	−0.60	0.04
des_12	3.14	0.67	−0.30	−0.27	0.04
des_13	3.05	0.72	−0.18	−0.73	0.04
des_14	2.96	0.75	−0.30	−0.32	0.04
des_15	3.08	0.68	−0.28	−0.25	0.04
des_16	3.03	0.66	−0.45	0.64	0.04
des_17	3.08	0.71	−0.50	0.26	0.04
des_18	3.17	0.67	−0.33	−0.29	0.04
IG	3.03	0.71	−0.29	−0.25	0.04

The results from the internal consistency analysis allow to consider the reliability assumption of the EDDPsic as acceptable. Adequate values were obtained for the Cronbach's alpha (α = 0.91), Rho standardized alpha (ρ = 0.92), and McDonald's omega (ω = 0.94) coefficients. In terms of the joint association degree among variables, adequate results were obtained in the KMO test (global MSA = 0.93) and the Bartlett sphericity test (χ^2^ = 2347.47; *p* = 0.000; *gl* = 153). This allows to validate the assumption that variables are correlated with each other.

The average age of the student body is associated with the level of advancement of studies (*r* = 0.45), in other words, students with lower average age are located in the lower academic cycles and those with higher age, in the higher cycles. In 4th cycle the average age was 20.26 with *SD* = 1.57, in 6th cycle the average age was = 21.75 with *SD* = 3.42, in 8th cycle the average age was 21.47 and *SD* = 1.74, and in 10th cycle the average age was = 23.53 and *SD* = 1.79. As can be seen, the lowest average age was in the 4th cycle and the highest average age was in the 10th cycle.

### Confirmatory Factor Analysis

**Figure 1:**
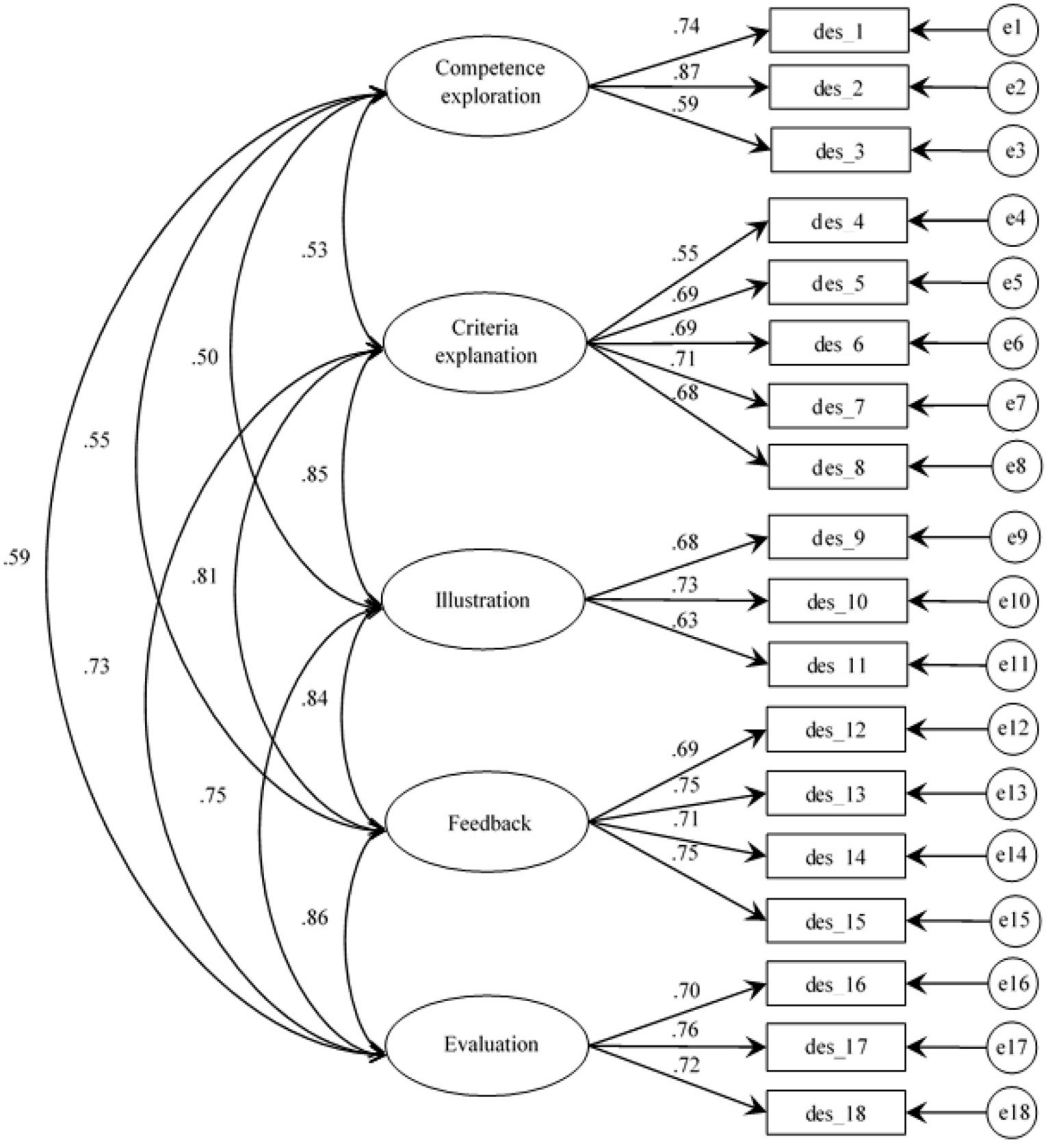
First order CFA five-factor model of the EDDPsic.

The fitness of the five-factor model was evaluated using *a-priori* established criteria. The analysis revealed that the five-factor model shows adequate fitness indices (χ^2^ = 229.29; *gl* = 125; CFI = 0.95; NNFI = 0.94; RMSEA = 0.052; SRMR = 0.050). Most of the factors yielded high and significant correlation values among them (see Figure 1). Factors with the highest correlation are F4 (Feedback) and F5 (Evaluation) with an *r* = 0.862 value; factors with the lowest correlation are F1 (Competence exploration) and F3 (Illustration) with an *r* = 0.502 value. Furthermore, standardized factorial loads of the five-factor model showed significant and adequate values. In the five-factor model, standardized variances of items ranged between 0.235 (des_2) and 0.697 (des_4).

### Factorial Invariance

A factorial invariance analysis was conducted through an MGCFA with the purpose of assessing whether the participants conceptualize the five-factor model equally, and thus be able to make valid comparisons among mean scores from the groups. Variables considered were age, gender, and academic stage, and participants were so divided into separate groups. Results obtained revealed adequate fitness evidence ($\chi_{p}^{2}$ > 0.05, ΔCFI < 0.01, RMSEA ≤ 0.06) for the configurational model in each of the three variables, so the model structure was considered to be the same for each group. Likewise, factorial invariance evidence was obtained for the weak (M1), strong (M2), and strict (M3) models in the age (M1: ΔCFI = −0.004; M2: ΔCFI = −0.001; M3: ΔCFI = −0.001) and gender variables (M1: ΔCFI = −0.001; M2: ΔCFI = −0.001; M3: ΔCFI = −0.001), so factorial loads, item intercepts and error variance were the same among all compared groups. In the case of the academic stage variable groups, invariance evidence was obtained for the weak and strong models (M1: ΔCFI = −0.004; M2: ΔCFI = −0.000) but not for the strict model (M3: ΔCFI = −0.018). Full results are shown in Table 4.

**Table 4:** Fitness indices for assessing the factorial invariance of the EDDPsic.

**Variable**	**Model**	***χ* ^2^**	**Δ*χ*^2^**	***p***	***gl***	**NNFI**	**CFI**	**ΔCFI**	**RMSEA**	**ΔRMSEA**
Age	Configurational	412.35	–	–	250	0.915	0.931	–	0.065	–
	Weak	433.93	21.58	0.06	263	0.915	0.927	−0.004	0.065	0.000
	Strong	448.27	14.33	0.35	276	0.918	0.926	−0.001	0.064	0.003
	Strict	468.29	20.02	0.33	294	0.922	0.926	−0.001	0.063	0.004
Sex	Configurational	419.73	–	–	250	0.911	0.927	–	0.067	–
	Weak	433.93	14.20	0.36	263	0.915	0.927	−0.001	0.065	0.004
	Strong	448.27	14.33	0.35	276	0.918	0.926	−0.001	0.064	0.003
	Strict	468.29	20.02	0.33	294	0.922	0.926	−0.001	0.063	0.004
Academic stage	Configurational	422.61	–	–	250	0.904	0.921	–	0.068	–
	Weak	444.48	21.87	0.057	263	0.904	0.917	−0.004	0.067	0.000
	Strong	458.38	13.90	0.381	276	0.908	0.917	0.000	0.066	0.004
	Strict	516.40	58.02	4.2e-6^*^	294	0.895	0.899	−0.018	0.071	−0.013

*According to Cheung and Rensvold (2002) ΔCFI ≤ −0.01 and according to Vandenberg and Lance (2000) a RMSEA ≤ 0.06 implies that the invariance assumption still holds*.

* *p ≤ 0.05*.

### Differences Among Groups

Statistical analyses were applied with the purpose of verifying the differences of IG scores of EDDPsic among groups of the age, gender, and academic stage.

For age, results show a significant difference (*F* = 4.905; *p* = 0.027) between group participants aged 21 or younger (*x* = 53.71, *SD* = 8.01) and those aged 22 or older (*x* = 55.82, *SD* = 8.33). The η^2^ coefficient indicates a small-sized effect (η^2^ = 0.016; Funder and Ozer, 2019), which accounts for 1.6% of the total variance. In terms of gender, results obtained indicate a non-significant difference between groups (*F* = 0.849; *p* = 0.358), so it is inferred that the correlation between women (*x* = 54.82, *SD* = 8.47) and men (*x* = 53.84, *SD* = 8.09) and the Psychology professor's performance assessment is almost non-existent. Lastly, regarding academic stage, results from the ANOVA test reveal significant differences (*F* = 34.24; *p* = 1.27e-8) between participants of the disciplinary stage (*x* = 52.07; *SD* = 7.5) and those from the terminal stage (*x* = 57.31, *SD* = 8.04). In addition, the η^2^ coefficient indicates a large-sized effect (η^2^ = 0.102; Funder and Ozer, 2019), which accounts for 32% of the total variance. ANOVA test results are shown in Table 5.

**Table 5:** ANOVA test of mean differences among *age, sex*, and *academic stage* groups.

**Variable**	**Condition**	**Sum of squares**	***gl***	**Root mean square**	***F***	**Sig**.	**η^**2**^**
Age	Among groups	325	1	325.2	4.905	0.027^*^	0.016
	Within groups	19,960	301	66.3			
Sex	Among groups	57	1	57.03	0.849	0.358	0.003
	Within groups	20,228	301	67.20			
Academic stage	Among groups	2,072	1	2071.9	34.24	1.27e-8^*^	0.102
	Within groups	18,213	301	60.5			

* *p ≤ 0.05*.

## Discussion and Conclusions

### Convergent and Divergent Construct Validity

One first aspect to highlight is that CFA results reveal a convergent and divergent validity of five constructs of didactic performance criteria and its 18 items grouped into the five factors, as established by the substantive theory on which the EDDPsic was based. The resulting CFA model confirmed the factorial structure for measurement of five didactic performance criteria (Bazán and Velarde, 2021). The factorial loads of each construct and its indicators (items) were acceptable (coefficients ranging between 0.59 and 0.87) and showed to converge adequately within each factor. On the other hand, findings derived from moderate and low covariances among constructs (0.53–0.86) confirmed that constructs diverge from one another.

By having students use this self-reporting scale, teaching behaviors can be identified in five didactic performance criteria according to the estimates of the substantive theory of this measurement. At a main level, four constructs of the five criteria or areas of teaching didactic performance initially proposed by Carpio et al. (1998) were confirmed. In addition, five of the seven criteria, as extended by Irigoyen et al. (2011) and Silva et al. (2014), were confirmed as well.

The results from the confirmatory factor analysis of this research match other findings in terms of validation and application of these categories for the analysis of didactic performance in several criteria or areas in middle high or higher teaching (Irigoyen et al., 2016; Velarde and Bazán, 2019; Bazán and Velarde, 2021). This means that it is possible to identify the didactic performance of Psychology teaching in at least five areas (criteria) with valid criteria: Competence Exploration, Criteria Explanation, Illustration, Feedback, and Evaluation. It is worth mentioning that these five areas of teaching didactic performance do not rule out the possibility of there being other areas forming didactic interactions, for example, the teaching–learning planning area or the practice supervision area among others.

The moderately high correlations between the Feedback and Evaluation criteria (0.86), and between the Criteria Explanation and Illustration (0.85) criteria are noteworthy. What elements can explain such associations? In the first case, findings from other research efforts have considered examinations, formative evaluation and feedback of students' performance as important elements within the learning assessment process (Marsh, 1984; Bell et al., 2019; Jellicoe and Forsythe, 2019; Krijgsman et al., 2019). This means that, although these two dimensions (Feedback and Evaluation) may be understood as categories that describe independent areas of didactic performance, both are related because they describe evaluation processes: the first one, referring to formative processes or in-progress attainment of achievement criteria and learning objectives; and the second one, as final evaluation of fulfillment of objectives and achievement criteria established for a determined term or period.

In the second case, the didactic performance areas Criteria Explanation and Illustration are two dimensions that might be correlated both conceptually and in the teaching practice itself. Other authors considered that both constitute the planning and organization characteristics of teaching (Marsh, 1984; García-Gómez et al., 2017; König et al., 2017; Nasser-Abu, 2017; Krijgsman et al., 2019). The performance area called Criteria Explanation corresponds to the planning of lessons and definition of objectives to be attained, whereas the Illustration performance area relates to the organization and development of the learning process. This last area of didactic performance of the professor implies that the professor demonstrates what students must do through actions and modeling (Ahn et al., 2020).

### Factorial Invariance in the Measurement of Didactic Performance Criteria

As previously stated, invariance analyses allow to control potential invalidity sources when measuring different properties of a variable, as they do in this study with five didactic performance criteria of the professor. One important aspect to consider in this study is that the factorial structure of the convergent and divergent validity of the five-criteria (performances) model was confirmed to be the same for the three control variables: age, sex, and academic stage, and with an adequate goodness-of-fit in all three cases.

In line with Marsh (1984) and Marsh and Hocevar (1984), the reported results reflect the multidimensional structure of the measurement of the professor's didactic performance (five dimensions or features), which is reliable and stable regardless of the age, sex, or academic stage group of the participants, and allows to identify and value the professor's performance as personal behaviors. Likewise, results reveal the possibility of generalizing the multivariate structure of the assessment of the professor's didactic performance according to the students for different age groups, for either men or women, and for different stages or course terms, or at different moments throughout the class or seminar. In addition, findings of this study underpin the importance of invariance analyses with multigroup approaches when measuring and validating multidimensional variables (Byrne, 2008; Milfont and Fischer, 2010; Hirschfeld and Von Brachel, 2014; Hornstein, 2017; Marsh et al., 2020).

On the other hand, the results for factorial invariance (weak, strong, and strict models) were the same for both the age control variable and the sex control variable. In both cases, an acceptable goodness-of-fit was obtained for the resulting models. This means that the structure, factorial loadings, item intercepts, and error variance of the structural model are similar for the groups belonging to both control variables and allow valid comparisons to be made. The MGCFA results show that the identification of five criteria of the professor's didactic performance according to the students is invariant in terms of age and sex. The variable sex, have proved to be good control sources for invariance in the students' assessment of teaching performance in universities (Marsh, 1984; Boring, 2015; Eouanzoui and Jones, 2017; Müller et al., 2017).

Furthermore, the education level or academic stage (current term attended by the undergraduate student) showed partial invariance evidence (only in the weak and strong models). The academic stage level (educational level) of the students has been referred to as an important factor to assess the measurement invariance of the didactic performance of professors, according to the assessment from students (Marsh, 1984; Marsh and Hocevar, 1984; Kalender, 2015).

### Differences Among Groups

When analyzing the differences among groups in the sex, age, and academic stage variables, significant differences were found only for the last two variables. This could mean that the scale to assess the didactic performance in five areas allows to identify differences according to the age and the academic stage groups of the students (disciplinary stage or final stage). However, sex seems not to be a variable that differentially affects the assessment of Psychology teaching performance.

One aspect to keep in mind is that these five teaching performance criteria refer to the didactic competencies that teachers display in their interaction with students in class or in practice, and these can be evaluated differently according to the stage of advancement in the professional training of the student being evaluated. The data from this study regarding differences by stage or academic cycle coincide with findings reported in studies on student evaluation, teaching and teacher performance in the context of higher education (Marsh and Hocevar, 1984; Kamran et al., 2012; Kalender, 2015; Müller et al., 2017; Kitto et al., 2019; Mocanu et al., 2021; Pérez-Villalobos et al., 2021). Furthermore, these differences in student perception may be related to student expectations and prior interest (van de Grift et al., 2016; Feistauer and Richter, 2018).

On the other hand, the age of the student body is a variable that seems to be an important indicator to identify differences in the student body's assessment of teaching and didactic performance of the teaching staff. Its effect may be closely related to the academic stage or cycle. Further multilevel analyses, controlling for the stage of studies variable, will be necessary to see the effect of student age on student ratings of teaching performance.

In this study, no significant differences were found according to sex of Peruvian psychology students, in the assessment of the didactic performance of the teacher in classes and practices at the undergraduate level in psychology, contrary to what has been reported when what is measured is the performance of the teacher in the traditional performance evaluation and in more varied samples according to career of origin (Boring, 2015; Boring et al., 2016; Eouanzoui and Jones, 2017). However, this situation may be linked to the disproportionate size of the female sample contrary to the reduced size of the male sample.

Based on this finding, it is pertinent to consider that the current literature on student evaluation of teaching and teacher performance emphasizes the inclusion of the student's gender variable in order to make a fairer and more equitable. Students may have biases in their assessment depending on whether they are female or male when evaluating the performance of their teachers, in which the female teacher has also been more affected (Potvin and Hazari, 2016; Heffernan, 2021; Kreitzer and Sweet-Cushman, 2021). According to Valencia (2021), this situation may encourage students to accept and reproduce these gender biases in other social contexts. However, Kreitzer and Sweet-Cushman (2021) point out that the effect of student gender on teaching evaluation is conditioned to other factors.

### Limitations

While it is true that the ratio between the number of participants in this study (316) and the number of items (18) is acceptable and sufficient to test the validity and reliability of a scale with confirmatory factor analysis when the items and constructs are derived from a substantive theory (Bentler and Chou, 1987; Bentler and Yuan, 1999; Thompson, 2000; Herzog and Boomsma, 2009), the sample size may contain some limitation, especially when the invariance analysis was performed controlling for the sex of the student body, since there was a disproportion in the size between the female and male samples. This disproportion in the size of the samples may be mainly due to the fact that the majority of the psychology student population is female. This disproportion will also be reflected in the gender samples.

However, it cannot be omitted to consider in studies of student perception, the sex variable as a possible source of invariance of the measures and differences between males and females (Boring, 2015; Eouanzoui and Jones, 2017), regarding the assessment of the didactic performance of their professors. Future studies on invariance of the measure on performance criteria based on the sex of the student body will have to consider larger samples in order to have a greater number of male students, although the disproportion by sex would still be present. Another option would be online applications of the instruments, which could also increase the sample size because the students could complete the instruments in the time most convenient to their interests and activities.

Another limitation of this study was the type of sampling, due to the fact that in these public universities from which the sample was taken, three factors are combined: (1) the authorization of the faculty council composed of representatives of the faculty, and a third of student representatives and administrative workers, which, based on the report on the pertinence of the research and ethical considerations, authorizes the realization of the research in its dependence, (2) the acceptance of the teacher of the subject in which the evaluation was made in person, and (3) the voluntary acceptance of each of the students to answer the self-report instruments in the selected subject and in which their teacher has accepted to carry out the study.

Thus, although the research project has the endorsement of the Faculty Council of both institutions, and a representative sample is expected in the number of subjects by stages of academic progress (study cycle), it was affected by the other two criteria. Likewise, a possible random selection of students to participate could leave out the representative distribution of subjects by cycle and stage of studies. Neither do these two universities have mechanisms for granting credits or any additional academic reward to guarantee a better recruitment process for participants.

A third limitation of the study is that the invariance analyses of the measure of the five criteria of teacher performance based on the stage of academic advancement of the students were made with measures of the evaluation of students in the intermediate and final stages, and invariance analyses have not been tested considering the different stages of academic training of the psychologist, for example, initial, intermediate and final stage (exercise of professional skills). Nor has the effect between progressive stages been measured, even including other stages of psychologists' academic progress, e.g., postgraduate degree (specialization, masters, doctorate). One aspect that should be considered for future research is to test models that assess the effect that the course progress has when students from later semesters are included, for example, undergraduate students from the terminal stage or postgraduate students.

### Conclusions

The findings derived from the confirmatory analyses carried out in the study reported here provided strong support for our expectations about the factorial structuring of the five criteria of teacher didactic performance in psychology classes, measured from the self-report of Peruvian students from two faculties of public universities, from different academic cycles of the psychology career. Consequently, a first aspect to conclude is that, the Scale of Teaching Performance of the Psychology Professor (EDDPsic) has proven to be a brief and reliable tool with an adequate construct validity to identify the professors' behavior in five theoretically determined areas of didactic performance in Psychology teaching (Carpio et al., 1998; Irigoyen et al., 2011, 2016; Silva et al., 2014).

A second aspect to conclude is that the results in this work showed measurement invariance among students according to age, sex, and level of advancement in their studies (intermediate and final stage) with respect to their evaluation of the didactic performance of their teachers, in five criteria or dimensions. The measurement of didactic performance in five dimensions (performance criteria or areas) is relatively invariant in terms of age, sex, and academic stage of the participants.

A third conclusion is that significant differences in the measurement of the professor's didactic performance areas depend on age differences and the undergraduate course progress of the students. The students' sex does not have any significant effects on their assessment of their Psychology professors' performance.

## Data Availability Statement

The raw data supporting the conclusions of this article will be made available by the authors, without undue reservation.

## Ethics Statement

Ethical review and approval was not required for the study on human participants in accordance with the local legislation and institutional requirements. The patients/participants provided their written informed consent to participate in this study.

## Author Contributions

AB-R contributed to the idea of research, its conceptualization, implementation, and methodology. He was in charge of writing the manuscript. He also contributed to the analysis and interpretation of data, and to the revision of the English version and writing in Frontiers format. JP-M directed the analysis and interpretation of data, contributed to the conceptualization of the research, and to the writing of the manuscript. He was also in charge of the revision of the English version and the writing in Frontiers format. BB-B collaborated in the analysis and interpretation of data, supported the search for additional bibliographic information, and review of the style of the article. All authors contributed to the article and approved the submitted version.

## Funding

This study was financed by a fellowship granted to AB-R as a researcher in Research Projects with Determined Resources—CANON-2018 of the Federico Villarreal National University—Public funds, Project RR 3479-2018-UNFV, led by Dr. Julio Inga Aranda.

## Conflict of Interest

The authors declare that the research was conducted in the absence of any commercial or financial relationships that could be construed as a potential conflict of interest.

## Publisher's Note

All claims expressed in this article are solely those of the authors and do not necessarily represent those of their affiliated organizations, or those of the publisher, the editors and the reviewers. Any product that may be evaluated in this article, or claim that may be made by its manufacturer, is not guaranteed or endorsed by the publisher.
