# Supplementary material for: Criteria for Teaching Performance in Psychology: Invariance According to Age, Sex, and Academic Stage of Peruvian Students
Source: Front Psychol. 2021 Oct 26;12:764081. doi: 10.3389/fpsyg.2021.764081 (PMC8589038; doi:10.3389/fpsyg.2021.764081)
Supplement: Supplementary file 1 [file Table_1.pdf]

Supplementary Table S1. Polychoric correlation matrix

	des_1	des_2	des_3	des_4	des_5	des_6	des_7	des_8	des_9	ds_10	ds_11	ds_12	ds_13	ds_14	ds_15	ds_16	ds_17	ds_18
des_1	1																	
des_2	0.76	1																
des_3	0.46	0.59	1															
des_4	0.38	0.44	0.42	1														
des_5	0.32	0.38	0.31	0.56	1													
des_6	0.31	0.37	0.29	0.46	0.68	1												
des_7	0.26	0.36	0.33	0.43	0.55	0.54	1											
des_8	0.26	0.36	0.31	0.31	0.53	0.57	0.67	1										
des_9	0.26	0.33	0.44	0.49	0.47	0.47	0.57	0.51	1									
des_10	0.33	0.39	0.38	0.44	0.54	0.49	0.56	0.54	0.62	1								
des_11	0.21	0.32	0.33	0.41	0.41	0.44	0.4	0.42	0.46	0.59	1							
des_12	0.29	0.45	0.33	0.45	0.43	0.52	0.46	0.49	0.46	0.46	0.52	1						
des_13	0.29	0.41	0.41	0.48	0.58	0.5	0.54	0.52	0.51	0.61	0.53	0.69	1					
des_14	0.35	0.45	0.4	0.35	0.37	0.45	0.49	0.47	0.48	0.48	0.42	0.54	0.61	1				
des_15	0.29	0.39	0.37	0.42	0.46	0.47	0.57	0.59	0.54	0.57	0.5	0.59	0.63	0.73	1			
des_16	0.31	0.36	0.43	0.5	0.38	0.43	0.45	0.46	0.48	0.44	0.46	0.47	0.55	0.6	0.58	1		
des_17	0.36	0.45	0.49	0.46	0.35	0.47	0.45	0.44	0.47	0.49	0.48	0.62	0.54	0.58	0.57	0.65	1	
des_18	0.44	0.45	0.43	0.5	0.53	0.45	0.44	0.42	0.46	0.45	0.43	0.52	0.58	0.53	0.53	0.59	0.65	1
